# Feasibility and Reproducibility of T2 Mapping Compared with Diffusion-Weighted Imaging in Solid Renal Masses

**DOI:** 10.3390/bioengineering11090901

**Published:** 2024-09-07

**Authors:** Shichao Li, Mengmeng Gao, Kangwen He, Guanjie Yuan, Ting Yin, Daoyu Hu, Zhen Li

**Affiliations:** 1 Department of Radiology, Tongji Hospital, Tongji Medical College, Huazhong University of Science and Technology, Wuhan 430030, China; 15172541672@163.com (S.L.); gaomm22@hust.edu.cn (M.G.); kangwen_he@163.com (K.H.); ygjforever98@163.com (G.Y.); daoyuhutjh@163.com (D.H.); 2 MR Collaborations, Siemens Healthineers Ltd., Chengdu 610041, China; ting.yin@siemens-healthineers.com

**Keywords:** clear cell renal cell carcinoma, quantitative MRI, T2 mapping, magnetic resonance imaging

## Abstract

Accurate prediction of renal mass subtypes, along with the WHO/ISUP grade and pathological T (pT) stage of clear cell renal cell carcinoma (ccRCC), is crucial for optimal decision making. Our study aimed to investigate the feasibility and reproducibility of motion-robust radial T2 mapping in differentiating lipid-poor angiomyolipoma (MFAML) from RCC and characterizing the WHO/ISUP grade and pT stage of ccRCC. Finally, 92 patients undergoing renal radial T2 mapping and ZOOMit DWI were recruited. The T2 values and apparent diffusion coefficient (ADC) were analyzed. Correlation coefficients were calculated between ADC and T2 values. Notably, ccRCC exhibited higher T2 and ADC values than MFAML (*p* < 0.05). T2 values were lower in the higher WHO/ISUP grade and pT stage of ccRCC (all *p* < 0.05). ADC showed no significant difference for pT stage (*p* = 0.056). T2 values revealed a higher area under the curve (AUC) in evaluating the WHO/ISUP grade compared to ADC (0.936 vs. 0.817, *p* = 0.027). T2 values moderately positively correlated with ADC (r = 0.675, *p* < 0.001). In conclusion, quantitative motion-robust radial T2 mapping is feasible for characterizing solid renal masses and could provide additional value for multiparametric imaging in predicting WHO/ISUP grade and pT stage of ccRCC.

## 1. Introduction

With the development and widespread availability of cross-sectional imaging techniques, the detection rate of incidental renal masses has dramatically increased. Up to 20% of solid small (<4 cm) renal masses are benign [1]. However, early therapeutic interventions for renal mass have not led to a reduction in tumor-specific mortality.

Before treating a solid renal mass, excluding a benign cause is essential to avoid unnecessary surgical intervention and loss of nephrons. Angiomyolipoma is the most common benign renal mass, and approximately 5% of cases do not show obvious fat content in conventional CT and MR images, a condition known as minimal fat AML (MFAML) [2]. Renal cell carcinoma (RCC) is the most common type of malignant renal tumor. The predominant pathological type is clear cell renal cell carcinoma (ccRCC), accounting for approximately 70% of RCCs [3]. Malignant tumors usually need surgical intervention, whereas benign masses may only require follow-up observation [4]. In addition, individualized treatment strategies are important due to the varying aggressiveness of ccRCCs. Those with a higher WHO/ISUP grade are more aggressive and associated with a more unfavorable prognosis [5]. Partial nephrectomy is recommended for small renal tumors. However, when a perirenal extension or vascular invasion occurs, the tumor is classified as pathological stage T3, for which open radical nephrectomy is the standard treatment [6]. Currently, preoperative evaluation primarily relies on percutaneous biopsy. However, due to the large heterogeneity of RCCs and the limited puncture tissue, tumor types can usually be distinguished, whereas pathological grades may not [7]. Ultimately, procedural complications may be unavoidable. Therefore, using noninvasive methods is imperative to accurately differentiate malignant renal tumors and predict the WHO/ISUP grade and pT stage of ccRCC.

Magnetic resonance imaging has been widely explored for the characterization of renal tumors and the prediction of the pathological gradings of ccRCC. Diffusion-weighted imaging (DWI) is considered a promising method. De Silva et al. [8] highlighted its effectiveness in stratifying RCC subtypes, while Ding et al. [9] demonstrated its potential in differentiating benign and malignant renal tumors. A meta-analysis further revealed its moderate diagnostic efficacy in differentiating the WHO/ISUP grades of ccRCC [10]. As a qualitative measurement method, T2-weighted (T2w) imaging provides good contrast for the identification of anatomical organ features in clinical settings, whereas the quantitative T2 mapping technique can reflect tissue composition, particularly free water content, and holds promise as a “noninvasive biopsy” method [11]. In recent decades, quantitative T2 values have been widely used for the diagnosis of myocardial and articular cartilage diseases [12,13], and also increasingly applied in tumor detection methods, such as prostate cancer and cervical squamous cell carcinoma imaging [14,15]. Adams first reported that lower-grade ccRCC exhibited significantly longer T2 relaxation times than higher-grade ccRCC [16]. Recently, Horvat-Menih et al. [17] also reported that T2 mapping showed a marked potential in quantifying differences across renal tumor subtypes and between ccRCC grades.

Conventional T2 measurements were primarily based on the multi-echo spin echo (MESE) method. However, this technique is motion-sensitive and requires a longer acquisition time, leading to limited spatial resolution, which affects its accuracy in characterizing pathological changes. Therefore, there is an urgent need for the exploration of rapid T2 mapping scanning techniques in clinical practice. The “pseudo” golden angle ratio (pGA) has been introduced as a novel view-ordering algorithm in 2D radial turbo spin echo (TSE). With this algorithm, the 2D radial TSE can support any ETL and reduce the scanning time without compromising the T2 mapping quality [18,19].

This study aimed to quantitatively evaluate the clinical utility of the motion-robust radial TSE T2 mapping sequence in differentiating MFAML from RCC and characterizing the WHO/ISUP grade and pT stage of ccRCC. We also compared the diagnostic performance of the T2 mapping sequence with traditional DWI to identify appropriate imaging methods and biomarkers, providing support for optimal treatment decision-making.

## 2. Materials and Methods

### 2.1. Patients

**Figure 1:**
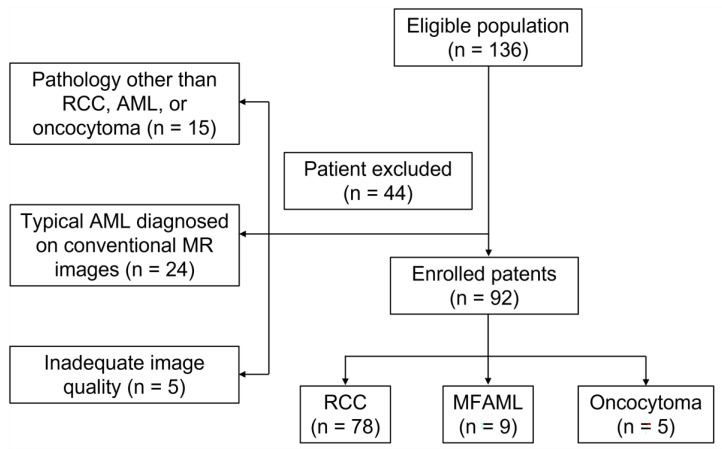
Flowchart of the patient population.

The local institutional review board (IRB) approved this retrospective study and waived informed consent requirements. Between June 2021 and April 2022, 136 patients with suspected renal tumors based on MRI screening were recruited for this study. The inclusion criteria were patients who received MRI examinations including radial TSE T2 mapping and ZOOMit DWI and underwent needle biopsy or resection with the postoperative pathological confirmation of RCC, MFAML, or oncocytoma. The exclusion criteria were as follows: (1) histopathological confirmation of pathologies other than RCC, MFAML, or oncocytoma (n = 15); (2) typical AML diagnosed on conventional MR images (n = 24); and (3) poor image quality (n = 5). Ultimately, 92 patients were enrolled in this study. The flowchart is displayed in Figure 1.

### 2.2. Imaging Acquisition

All MRI scans were performed on a 3.0 Tesla magnet with an 18-channel body phased-array receiver coil. All participants underwent scanning in a feet-first supine position. A prototype free-breathing 2D radial TSE sequence was used for T2 mapping, which is more motion-robust than conventional cartesian acquisitions due to the radial k-space trajectory. The T2 values were generated using the slice-resolve extended-phase graph model with a dictionary-based algorithm [20]. Axial ZOOMit DWI applies both echo-planar imaging and another parallel radiofrequency pulse sequence to obtain a zoomed field-of-view (FOV) that only covers the region of interest (ROI) and consequently reduces geometric distortion and susceptibility artifacts, which allows for better image quality and provides more anatomical detail [21]. The apparent diffusion coefficient (ADC) map was calculated using a monoexponential model. The scan parameters are summarized in Table 1.

### 2.3. Image Analysis

To obtain the mean T2 maps and ADC values of the tumor, its region-of-interest (ROI) was manually delineated by two radiologists (with two and seven years of experience in abdominal MRI diagnosis) who were blinded to the clinical and pathological information. For both maps, a circular ROI was delineated around the entire tumor. When a necrotic or cystic component was noted inside the tumor, a circular 2D ROI was placed on the most homogeneous portion of the solid area and set to the largest possible area using the T2-weighted image as a reference [16]. One radiologist repeated the ROI delineation after two weeks. 

### 2.4. Statistical Analysis

All the statistical analyses were performed in SPSS (version 26.0, IBM SPSS, Chicago, IL, USA) and GraphPad Prism software (version 8.3.0, GraphPad Software Inc., San Diego, CA, USA). The Shapiro–Wilk test was used to estimate the normality of the data distribution. Age, tumor size, T2 values, and ADC values were averaged across measurements and expressed as means ± standard deviation (SD) for normally distributed data or medians (interquartile range, IQR) in the absence of normal distribution. The Mann–Whitney U test was used to evaluate the differences. In addition, the intra- and interobserver variabilities of T2 mapping and DWI measurements were evaluated using intraclass correlation coefficients and Bland–Altman plots. Parameters with *p* < 0.05 were included for a forward stepwise binary logistic regression analysis to determine the best test combination, and a probability value was generated. Receiver operating characteristic (ROC) curve analysis was performed, and the area under the ROC curve (AUC) was calculated to evaluate the diagnostic performances. AUCs of different parameters and combinations were compared using DeLong’s test. Lastly, Spearman’s rank correlation analysis was used to correlate the ADC and T2 values.

## 3. Results

### 3.1. Histopathological Findings

The final study population comprised 92 patients (59 men and 33 women, mean age, 54.4 ± 11.9; age range, 23–78 years) with histologically diagnosed RCC (n = 78), MFAML (n = 9), and oncocytoma (n = 5). There were 67 cases of ccRCC, 4 of which provided no grading or staging results due to needle biopsy. Histologic examinations revealed 12 WHO/ISUP grade I lesions, 36 WHO/ISUP grade II lesions, 9 WHO/ISUP grade III lesions, and 6 WHO/ISUP grade IV lesions. Of the 63 patients, 50 were in the lower pT stage group (pT1 and pT2 stage), and the remaining 13 were in the higher pT stage group (pT3 and pT4 stage). The maximum ccRCC diameter was determined in axial T2-weighted images, between 1.5 cm and 12.7 cm. The demographic and clinicopathological data of the 92 patients are listed in Table 2.

### 3.2. Intra- and Interobserver Agreement

**Figure 2:**
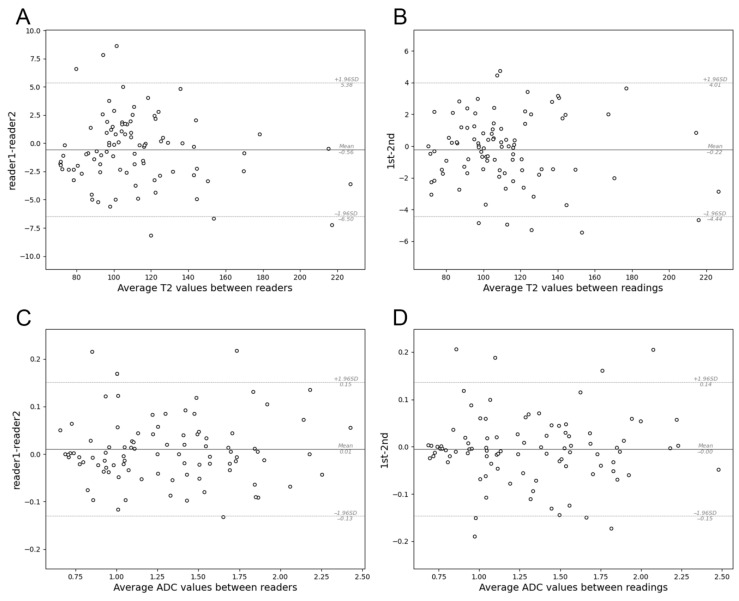
The Bland–Altman plots of apparent diffusion coefficients (ADCs) and T2 values for 92 renal masses: comparison of T2 values measured by (**A**) two different observers and (**B**) the same observer; comparison of ADCs measured by (**C**) two different observers and (**D**) the same observer.

Intraclass correlation coefficients (ICCs) revealed excellent intra- and interobserver agreement for the T2 values (ICC, 0.997; 95% CI, 0.997–0.998, and ICC, 0.999; 95% CI, 0.998–0.999, respectively) and ADC (ICC, 0.993; 95% CI, 0.989–0.995, and ICC, 0.993; 95% CI, 0.989–0.995, respectively). In the Bland–Altman plots, points in each plot tended to cluster around the mean difference line (Figure 2). Due to the remarkable agreement between the mean T2 and ADC values obtained by the two radiologists, the mean of three measurements was used for further analysis.

### 3.3. T2 Mapping and DWI Results of Different Tumor Pathological Types and Grades

**Figure 3:**
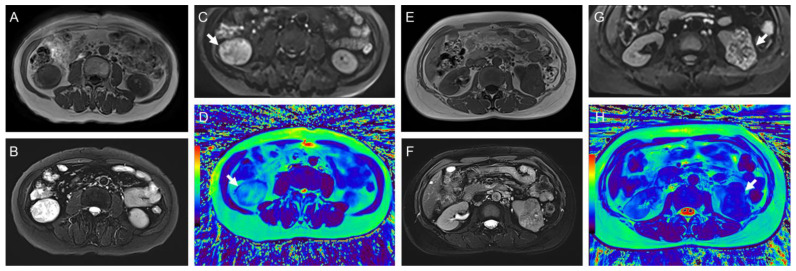
(**A**–**D**) Representative images of a 65-year-old woman with WHO/ISUP II ccRCC in the right kidney (arrow): (**A**) axial T1-weighted image; (**B**) T2-weighted image; (**C**) diffusion-weighted images (b = 800 s/mm^2^); (**D**) radial TSE T2 map. The mean ADC and T2 values of the tumor were 1.51 mm^2^/s and 141.4 ms, respectively. (**E**–**H**) Representative images of a 51-year-old man with WHO/ISUP III ccRCC with tumor necrosis in the left kidney (arrow): (**E**) axial T1-weighted image; (**F**) T2-weighted image; (**G**) diffusion-weighted images (b = 800 s/mm^2^); (**H**) radial TSE T2 map. The mean ADC and T2 values of the tumor were 1.11 mm^2^/s and 85.9 ms, respectively. The color bar in the pseudocolor images (D and H) represents T2 values, which gradually increase from black to red.

**Figure 4:**
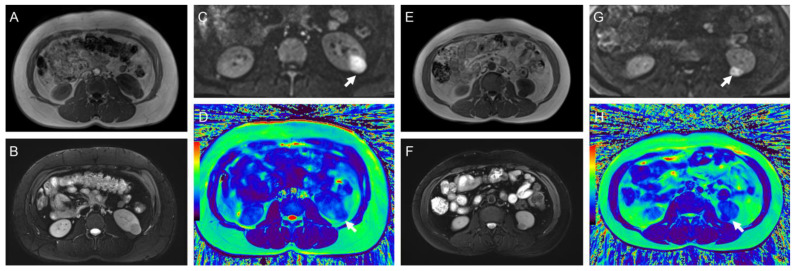
(**A**–**D**) Representative images of a 44-year-old woman with minimal fat angiomyolipoma in the left kidney (arrow): (**A**) axial T1-weighted image; (**B**) T2-weighted image; (**C**) diffusion-weighted images (b = 800 s/mm^2^); (**D**) radial TSE T2 map. The mean ADC and T2 values of the tumor were 0.69 mm^2^/s and 82.2 ms, respectively. (**E**–**H**) Representative images of a 44-year-old man with chromophobe RCC in the left kidney (arrow): (**E**) axial T1-weighted image; (**F**) T2-weighted image; (**G**) diffusion-weighted images (b = 800 s/mm^2^); (**H**) radial TSE T2 map. The mean ADC and T2 values of the tumor were 1.08 mm^2^/s and 116.0 ms, respectively. The color bar in the pseudocolor images (D and H) represents T2 values, which gradually increase from black to red.

**Figure 5:**
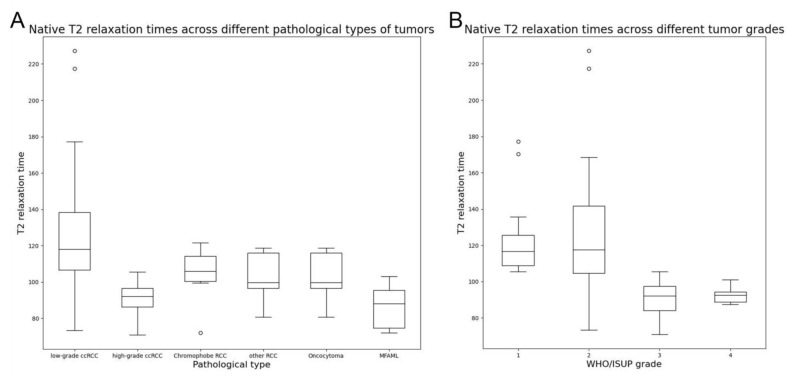
The distribution of T2 values across (**A**) different pathological types of tumors and (**B**) different WHO/ISUP grades of clear cell renal cell carcinomas.

The typical multiparameter MR images of different pathological types and WHO/ISUP grades are illustrated in Figure 3 and Figure 4, and the distribution of T2 and ADC values is provided in Table 3 and Figure 5. 

Regarding the differentiation of benign and malignant renal tumors, significant differences were observed in the T2 and ADC values between malignant and benign tumors, as well as between ccRCC and MFAML. The T2 and ADC values of malignant renal tumors were significantly higher than those of benign renal tumors (*p* = 0.002, *p* = 0.016, respectively), and higher T2 and ADC values were observed in ccRCC than in MFAML (*p* < 0.001, *p* < 0.001, respectively). 

Considering the WHO/ISUP grading, significant differences were observed in the T2 and ADC values of ccRCC among different WHO/ISUP grades (*p* < 0.001, *p* < 0.001, respectively). Tumors with a higher WHO/ISUP grade yielded significantly lower T2 values (90.04 ± 9.20 vs. 125.10 ± 29.82), consistent with ADC values. 

In terms of pT staging, the T2 values of ccRCCs at higher pT stages were significantly lower than those of ccRCCs at lower pT stages (*p* = 0.002), whereas their ADC values did not differ significantly (*p* = 0.056).

### 3.4. Diagnostic Efficiency and Comparison

**Figure 6:**
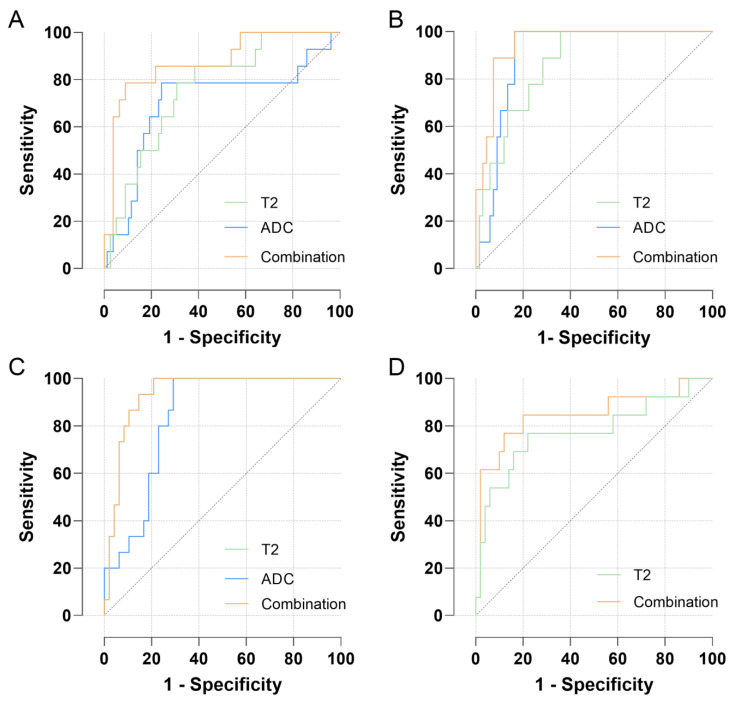
The receiver operating characteristic (ROC) curves for discriminating (**A**) malignant and benign renal masses, (**B**) clear cell renal cell carcinoma and minimal fat angiomyolipoma, (**C**) different WHO/ISUP grades, and (**D**) pT stages of clear cell renal cell carcinoma.

The ROC curves of the T2 mapping and DWI for distinguishing malignant and benign tumors and predicting the WHO/ISUP grade and pT stage are presented in Table 4 and Figure 6. 

In terms of the differentiation of malignant and benign renal tumors, the AUCs of the T2 and ADC values were 0.761 and 0.702, respectively. The combination of age, tumor size (maximum tumor diameter), and T2 values yielded the highest AUC of 0.875, which was significantly higher than that of either T2 or ADC values alone (*p* = 0.003 and *p* = 0.004, respectively). 

Regarding the differentiation of ccRCC and MFAML, the AUCs for the T2 and ADC values were 0.862 and 0.900, respectively. Based on binary logistic regression analysis, age and ADC values were included in the combination with the highest AUC value of 0.949; however, no significant differences were observed between this combination and T2 or ADC values alone (all *p* > 0.05). 

For the prediction of the WHO/ISUP grade, the AUCs for the T2 and ADC values were 0.936 and 0.837, respectively, indicating a significant difference (*p* = 0.027). 

For the prediction of the pT stage, the AUC value of the T2 values was 0.775, whereas the ADC values revealed no significant difference. Combining tumor size with T2 values yielded the highest AUC of 0.848, but no significant difference was observed between the combination and the T2 value alone (*p* > 0.05). 

In addition, a moderate positive correlation was observed between the T2 and ADC values (r = 0.675, *p* < 0.001). 

## 4. Discussion

Currently, incidental detection of renal masses has been rising steadily. Early therapeutic interventions have no obvious effect on cancer-specific mortality, and the preservation of renal function is essential for the long-term survival of patients. Therefore, exploring potential noninvasive imaging tools for accurate preoperative diagnosis is necessary for optimal management. Our study revealed that T2 mapping can be used to differentiate pathological types of renal masses and predict ccRCC’s pathological grade and stage. In addition, our study found that T2 values demonstrated better diagnostic performance than ADC in predicting the WHP/ISUP grade, highlighting the potential of T2 mapping as a noninvasive biomarker for renal masses.

Conventional T2-weighted imaging can only be used for qualitative analysis and have arbitrary signal intensity scales [16]. Crombé et al. [22] reported that T2 mapping provided better repeatability, reproducibility, and performance of radiomics than T2WI. T2 relaxation time depends on the tissue composition, such as macromolecular concentration and tissue water content, whereas quantitative T2 mapping can reflect pathological changes in the tissue composition [23]. Initial T2 mapping efforts have primarily focused on myocardial and cartilage imaging [13,24,25]. However, T2 mapping can also be applied to other organs, such as the breast, liver, and kidney [23,26,27,28], considering tissue fibrosis and tumor malignancy. Gu et al. [29] revealed that quantitative T2 mapping is feasible for the preoperative grading of gliomas. Adams et al. [16] first reported that T2 relaxation times have the potential to noninvasively assess the ccRCC tumor grade, with higher T2 values in low-grade ccRCC than in high-grade tumors, consistent with our study. 

To the best of our knowledge, ccRCC can exhibit varying degrees of internal necrosis, hemorrhage, and cystic changes, which are closely associated with tumor grading and prognosis. Low-grade ccRCCs typically present with cystic changes, while intratumoral necrosis is particularly common in higher-grade ccRCCs and has been shown to be an independent marker of poor prognosis [20]. These histopathological changes may explain the more prolonged relaxation times observed in low-grade ccRCCs compared to high-grade ccRCCs in our study. Moreover, the presence of densely packed proliferating cells, interstitial reticulin deposition, and/or irregular tumor vasculature within higher-grade ccRCCs contributes to the decrease in T2 values. However, several cases of low-grade ccRCCs exhibited lower T2 values, possibly due to micronecrosis within the tumor [16]. A meta-analysis concluded that DWI can be used to differentiate the WHO/ISUP grades [10], which was considered a quantitative reference biomarker in this study. In contrast to ADCs, T2 values yielded significantly higher AUC values. A moderate positive correlation was also observed between the ADC and T2 values. Langer et al. [30] demonstrated that the ADC and T2 values are both linked to cell density. ADC reflect the diffusion motion of water molecules, which is primarily affected by the cell density and microstructure, whereas T2 is primarily affected by the water content, the state of water (e.g., the fraction of free water vs. macromolecule-bound water, fluid shifts between extracellular and intracellular compartments, intracellular vacuolation, collagen content, and the orientation of collagen fibrils), and the local macromolecular environment [31,32].

This study also further verified the feasibility of T2 mapping in differentiating benign and malignant renal masses and the pathological staging of ccRCC. Tanaka et al. [33] reported that younger age was associated with MFAML, consistent with our study. The T2 value combined with age and tumor size (maximum tumor diameter) significantly improves the ability to distinguish benign from malignant renal masses. Considering the prediction of the pT of ccRCC, combining tumor size with the T2 value showed a higher AUC compared to using the T2 value alone (AUC: 0.848 vs. 0.775); however, the difference was not statistically significant (*p* > 0.05). Tumor size correlates with renal sinus and peri-renal invasion and vena cava infiltration [34].

In a previous study, Adams et al. [16] investigated a T2-prepared single-shot TrueFISP sequence. Although insensitive to respiratory motion artifacts, their results revealed a limited number of echoes that might affect the accuracy of T2 value calculations. The T2 mapping sequence in this study included a multi-shot radial turbo spin echo (TSE) technique. Multiple echoes were acquired using the pseudo golden-angle (pGA) radial reordering and restricted using a k-space view-sharing method to preserve the targeted TE contrasts [18,19]. Ti is motion-robust and suitable for abdominal imaging.

This study has several limitations. First, this was a retrospective and small-sample study of a single-institutional design, and our results may not be generalizable to other institutions. Second, ccRCC is the most common renal malignancy, and the sample size of other pathological types of renal masses in this study was small, which may pose a potential risk of selection bias. Third, the T2 values of the tumor were only measured in one representative axial plane, not in the whole tumor. 

## 5. Conclusions

Quantitative motion-robust radial T2 mapping holds promise as a noninvasive tool for characterizing solid renal masses and can provide additional value for multiparametric imaging in predicting the WHO/ISUP grade and pT stage of ccRCC, which is of great significance in guiding optimal decision-making.

## Figures and Tables

**Table 1:** MRI acquisition parameters.

	Morphological T2w FSE	DWI	T2 Mapping
Sequence	Spin-echo	EPI	Radio TSE
Repetition time (ms)	4630	7700	2100
Echo time (ms)	77	71	7.7
Slice thickness (mm)	4	4	5
Acquisition matrix	320 × 320	128 × 128	256 × 256
Field of view (mm)	380 × 380	125 × 288	300 × 300
No. averages	1	-	1
Bandwidth (Hz/Px)	781	1666	488
Radial views	-	-	320
b-values (s/mm^2^)	-	50/800	-
Phase encoding direction	R >> L	A >> P	A >> P
Acquisition duration (min:s)	-	2:30	1:30

MRI, magnetic resonance imaging; T2w, T2-weighted; EPI, echo planar imaging; FSE, fast spin-echo.

**Table 2:** Characteristics of the 92 patients with renal masses.

Parameter	Data
Age (y) *	54.4 ± 11.9
Gender	
male	59
female	33
Mean tumor diameter (cm) *	4.45 ± 2.27
Pathological type	
Clear cell RCC	67
Minimal fat angiomyolipoma	9
Chromophobe RCC	6
Oncocytoma	5
Papillary RCC	1
MiT family translocation RCC	1
Mucinous tubular and spindle cell RCC	1
Unclassified RCC	2
WHO/ISUP grade	
I	12
II	36
III	9
IV	6
Pathological T stage	
T1a	30
T1b	19
T2a	1
T3a	10
T3b	2
T4	1

* Data are mean ± standard deviation (SD).

**Table 3:** T2 values compared to different types of renal masses, WHO/ISUP grades, and pathological T stages.

Groups	T2 Values (ms)	ADC Values (×10^−3^ mm^2^/s)
Tissue type		
Malignant (n = 78)	115.00 ± 30.91 (96.91–125.79)	1.35 ± 0.41 (1.05–1.66)
Benign (n = 14)	92.13 ± 14.88 (79.27–100.51)	1.11 ± 0.45 (0.83–1.22)
*p*-value	0.002	0.016
Tissue type		
ccRCC (n = 67)	116.02 ± 29.59 (97.52–126.41)	1.39 ± 0.37 (1.08–1.68)
MFAML (n = 9)	86.46 ± 11.78 (73.44–97.34)	0.91 ± 0.11 (0.83–1.01)
*p*-value	<0.001	<0.001
WHO/ISUP grade		
High-grade (n = 15)	90.04 ± 9.20 (84.15–97.52)	1.10 ± 0.22 (0.96–1.26)
Low-grade (n = 48)	125.10 ± 29.82 (106.54–140.41)	1.51 ± 0.35 (1.28–1.74)
*p*-value	<0.001	<0.001
Pathological T stage		
High-stage (n = 13)	98.25 ± 23.35 (82.33–110.17)	1.22 ± 0.30 (1.05–1.55)
Low-stage (n = 50)	121.56 ± 30.26 (104.03–136.08)	1.46 ± 0.37 (1.22–1.72)
*p*-value	0.002	0.056

ccRCC, renal cell renal cell carcinoma; MFAML, minimal fat angiomyolipoma. T2 and ADC values are expressed as mean ± standard deviation, and the interquartile ranges are provided in parentheses.

**Table 4:** Diagnostic performance of T2 and ADC values.

Category	Threshold	AUC (95% CI)	Sensitivity	Specificity	*p* for Comparison
Malignant vs. benign					
T2 value (ms)	99.79	0.761 (0.639–0.883)	69.23%	78.57%	0.003
ADC (×10^−3^ mm^2^/s)	1.05	0.702 (0.530–0.875)	75.64%	78.57%	0.004
Combination (age + size + T2 value)	NA	0.875 (0.770–0.979)	91.00%	78.60%	Ref
ccRCC vs. MFAML					
T2 value (ms)	103.54	0.862 (0.766–0.959)	64.18%	100.00%	0.123
ADC (×10^−3^ mm^2^/s)	1.05	0.900 (0.832–0.969)	83.58%	100.00%	0.084
Combination (age + ADC)	NA	0.949 (0.899–0.999)	83.60%	100.00%	Ref
WHO/ISUP grade					
T2 value (ms)	105.56	0.936 (0.877–0.995)	100.00%	79.17%	1.000
ADC (×10^−3^ mm^2^/s)	1.39	0.837 (0.741–0.934)	100.00%	70.83%	0.027
Combined (T2 value)	NA	0.936 (0.877–0.995)	100.00%	79.17%	Ref
Pathological T stage					
T2 value (ms)	101.98	0.775 (0.608–0.943)	76.92%	78.00%	0.531
ADC (×10^−3^ mm^2^/s)	-	-	-	-	-
Combination (size + T2 value)	NA	0.848 (0.704–0.991)	76.90%	88.00%	Ref

ADC, apparent diffusion coefficient; AUC, area under the curve; CI, confidence interval; ccRCC, clear cell renal cell carcinoma; MFAML, minimal fat angiomyolipoma. Combinations were selected using binary logistic regression. Ref, reference. “Tumor size” refers to the maximum tumor diameter.

## Data Availability

The datasets used and/or analyzed during the current study are available from the corresponding author on reasonable request.
